# A review of the genus *Neopsocopsis* (Psocodea, “Psocoptera”, Psocidae), with one new species from China

**DOI:** 10.3897/zookeys.203.3138

**Published:** 2012-06-20

**Authors:** Lu-Xi Liu, Kazunori Yoshizawa, Fa-Sheng Li, Zhi-Qi Liu

**Affiliations:** 1 Department of Entomology, China Agricultural University, Beijing, 100193, China; 2 Systematic Entomology, Graduate School of Agriculture, Hokkaido University, Sapporo, 060-8589, Japan

**Keywords:** Psocodea, Psocoptera, Psocidae, *Neopsocopsis*, redescriptions, new synonymies, new species, new records, distribution, key

## Abstract

A review of species of the genus *Neopsocopsis* Badonnel, 1936 is presented. Four species are redescribed, viz. *Neopsocopsis hirticornis* (Reuter, 1893), *Neopsocopsis quinquedentata* (Li & Yang, 1988), *Neopsocopsis profunda* (Li, 1995), and *Neopsocopsis flavida* (Li, 1989), as well as the description of one new species, *Neopsocopsis convexa* **sp. n.** Seven new synonymies are proposed as follows: *Pentablaste obconica* Li **syn. n.** and *Pentablaste clavata* Li **syn. n.** of *Neopsocopsis hirticornis*, *Pentablaste tetraedrica* Li **syn. n.** of *Neopsocopsis longiptera*, *Neoblaste schizopetala* Li **syn. n.** and *Neoblaste flavae* Li **syn. n.** of *Neopsocopsis profunda*, *Blastopsocidus pini* Li **syn. n.** and *Pentablaste lanceolata* Li **syn. n.** of *Neopsocopsis flavida*. *Neopsocopsis hirticornis* (Reuter, 1893) is recorded from Japan and China for the first time, and *Neopsocopsis longiptera* Vishnyakova, 1986 is newly recorded from China. Illustrated keys to adult males and females are presented.

## Introduction

The psocopteran genus *Neopsocopsis* is a small group in the subfamily Amphigerontiinae, formerly characterized by head-covering glandular setae, female fore wing brachypterous, and male hypandrium with three lobes (one median lobe and two lateral lobes) and 2 internal apophyses (Badonnel 1936; Günther 1974; Smithers 1972). In 1986, Vishnyakova redefined the genus, pointing out the existence of macropterous female specimens. Afterward, Yoshizawa (2010) synonymized the Chinese genus *Pentablaste* Li with *Neopsocopsis* and considered the genus to be a well defined monophyletic group, mainly based on genitalic characters. The type species, *Neopsocopsis hirticornis* (Reuter, 1893), is widely distributed in the Palaearctic Region ranging from East Asia to West Europe, and the remaining bulk of species occurring in the Oriental Region, principally in Japan and China.

Badonnel (1935) described *Neopsocus pyrenaicus* based on a single female specimen collected from France, with the character states: 1) body-covering glandular setae and 2) a brachypterous fore wing. Later, in 1936, after reexamination of the accompanying male specimens collected with *Neopsocus pyrenaicus*, Badonnel separated *Neopsocopsis* from *Neopsocus* as a new genus on the basis of distinct male venational and genitalic characters, with *Neopsocopsis pyrenaicus* as the type species. In 1938, Badonnel moved the Finland species *Psocus hirticornis* Reuter, 1893 (=*Pentablaste bastmannianus* Enderlein, 1918) to *Neopsocopsis* and considered *Neopsocopsis pyrenaicus* as a subspecies of *Neopsocopsis hirticornis*, and later proposed it as a new junior synonymy (Badonnel, 1982). Afterward, macropterous females of *Neopsocopsis hirticornis* were found from Europe (Günther 1980, 1991; Hedström 1989). Meinander (1981) described a second species from Egypt, *Neopsocopsis aegyptiacus*, which was proposed as a junior synonym of *Blaste (Euclismiopsis) medleri* New by Lienhard (1986). Vishnyakova (1986) and Yoshizawa (2010) described *Neopsocopsis longiptera* (from Russia) and *Neopsocopsis sakishimensis* (from Japan), respectively. Yoshizawa (2010) also treated the Chinese genus *Pentablaste* Li, 2002 as a new junior synonym of the genus *Neopsocopsis*, which raised the species of the genus to 19.

In the present paper, one new species of *Neopsocopsis* is described,* N. convexa* sp. n., with redescriptions of four species: *Neopsocopsis hirticornis* (Reuter, 1893), *Neopsocopsis quinquedentata* (Li & Yang, 1988), *Neopsocopsis profunda* (Li, 1995), and *Neopsocopsis flavida* (Li, 1989). Seven new synonymies are proposed as follows: *Pentablaste obconica* Li, syn. n. and *Pentablaste clavata* Li, syn. n. of *Neopsocopsis hirticornis*, *Pentablaste tetraedrica* Li, syn. n. of *Neopsocopsis longiptera*, *Neoblaste schizopetala* Li, syn. n. and *Neoblaste flavae* Li, syn. n. of *Neopsocopsis profunda*, *Blastopsocidus pini* Li, syn. n. and *Pentablaste lanceolata* Li, syn. n. of *Neopsocopsis flavida*. *Neopsocopsis hirticornis* (Reuter, 1893) is recorded from Japan and China for the first time, and *Neopsocopsis longiptera* Vishnyakova, 1986 is newly recorded from China. Updated keys for adult males and females of world species in the genus is presented.

## Material and methods

All specimens treated in this paper were from Entomological Museum of China Agricultural University (CAU), Beijing, China, and Hokkaido University Insect Collection (SEHU), Sapporo, Japan. Specimen preparation and measurements were undertaken following Liu et al. (2011). Images of fore wings were taken with a Canon EOS 500D digital camera attached to a stereoscopic microscope.

## Taxonomy

### Neopsocopsis

Badonnel

http://species-id.net/wiki/Neopsocopsis

Neopsocopsis Badonnel, 1936: 420. Type species: *Psocus hirticornis* Reuter, 1893: 42, original designation.
Pentablaste Li, 2002: 1367. Type species: *Pentablaste obconica* Li, 2002. Synonymy: Yoshizawa, 2010: 24.

#### General characters.

Small to medium sized psocids. Antennae short, not reaching tip of fore wing. Wings membranous, usually hyaline with brownish tinge; fore wing normal in both sexes or brachypterous in female; fore wing Rs and M meeting at point, fused for short distance or connected by crossvein, areola postica pentagonous, first and second sections of Cu_1a_ forming obtuse angle about 120°. Male abdomen with distal segments dark brown colored, 8^th^ sternum broadly sclerotized and fused to hypandrium, with lateral margins overlapping clunium; epiproct round, dorsally with sclerotized projection at middle of anterior margin; hypandrium symmetrical and 5-lobed, with posteromedian lobe forming dorsal-curved structure, pair of lateral lobes carinate with outer surface covering denticles, and pair of internal lobes rod-like or expanded; phallosome free posteriorly, anteriorly fused or connected by membrane. Female subgenital plate with sclerotized arms forming flat V-shaped regions and expanded laterally, egg guide relatively long; ventral valve of gonapophyses distally tapering to slender tip, outer valve with well developed posterior lobe.

#### Distribution.

China; Finland; France; Germany; Hungary; Italy; Japan; Macedonia; Mongolia; Romania; Russia; Serbia; Spain; Sweden; Switzerland.

#### Remarks.

The genus *Neopsocopsis* is placed in the subfamily Amphigerontiinae mainly based on the following characters: male 8^th^ sternum broadly sclerotized and fused to hypandrium, with lateral margins overlapping clunium (Yoshizawa et al. 2011); hypandrium symmetrical with various projections; phallosome free posteriorly; female subgenital plate with prominent egg guide plate and ventral valve of gonapophyses tapering distally (also observed in some other genera of Psocinae). *Neopsocopsis* can be easily distinguished from genera in Amphigerontiinae by the 5-lobed hypandrium as well as the carinate and dentigerous lateral lobes, which are considered to be an autapomorphy of the genus. In addition, the shape and sclerotization pattern of the female subgenital plate are also distinct in Amphigerontiinae. Described based on a single male specimen from China, *Pentablaste pentasticha* (Li, 1990) apparently lacks the above characters, and it appears to correspond more closely to the generic characters of *Neoblaste* in genitalic details. However, classification of this species cannot be confirmed until more samples are analyzed, and we do not discuss *Pentablaste pentasticha* in this work.

One Indonesian genus, *Javablaste* Endang, Thornton & New, 2002, shared many generic characters of *Neopsocopsis* and was different from the latter by 1) female with normal fore wing, 2) subgenital plate with transverse sclerotized bar at mid line and 3) male hypandrium with lateral spinous lobes (Endang et al. 2002). However, as discussed above, the brachypterous fore wing is not a stable character of *Neopsocopsis*. Later, in 2010, Endang and New recorded three new species of *Javablaste* from Sumatra, Indonesia, including *Javablaste darmayasai* Endang & New, 2010, in which the second condition was not observed. In addition, there is little difference between the terms “tuberculate” or “spinous” in reference to the lateral lobes of the hypandrium. According to Endang and New (2010), the Chinese species, *Neopsocopsis flavida*, was pointed out to be very similar to speciesof *Javablaste* with minor genitalic differences. It is strong possible that *Javablaste* is also a junior synonym of *Neopsocopsis*.

## Identification keys

*Pentablaste pentasticha* (Li, 1990) is not included in the key as re-examination of the species and its possible relationship with *Neoblaste* were not possible for this study.

### Key to adult males of *Neopsocopsis*

**Table:** 

1	Internal lobes of hypandrium well developed, expanded and longer than posteromedian lobe (Fig. 3C)	2
–	Internal lobes of hypandrium not well developed, usually rod-like, equal length or shorter than posteromedian lobe (Fig. 4C)	3
2	Small in size, fore wing length about 2.5–3.0 mm; epiproct fully sclerotized, with tiny projection dorsally; lateral lobes of hypandrium small; phallosome with parameres absent. See Yoshizawa, 2010, Fig. 10	*Neopsocopsis sakishimensis*
–	Large in size, fore wing length about 3.2–4.5 mm; epiproct with membranous regions medially (Fig. 3B), with large and sharp projection dorsally (Fig. 3AB); lateral lobes of hypandrium large (Fig. 3C); phallosome paired with parameres (Fig. 3D)	*Neopsocopsis hirticornis* (=*Pentablaste obconica*; =*Pentablaste clavata*)
3	Posteromedian lobe of hypandrium with distal margin almost straight, or concave with projection medially (Fig. 5C)	4
–	Posteromedian lobe of hypandrium with distal margin convex medially (Fig. 2C)	6
4	Posteromedian lobe of hypandrium with distal margin straight; lateral lobes of hypandrium with anterior part short. See Yoshizawa, 2010, Fig. 12	*Neopsocopsis longiptera* (=*Pentablaste tetraedrica*)
–	Posteromedian lobe of hypandrium with distal margin concave, and with projection medially; lateral lobes of hypandrium with anterior part long and curved anteromedially (Fig. 4C)	5
5	Epiproct with sharp projection dorsally (Fig. 4B); internal lobes of hypandrium not forked distally (Fig. 4C)	*Neopsocopsis quinquedentata*
–	Epiproct with large and round projection dorsally (Fig. 5B); internal lobes of hypandrium forked distally (Fig. 5C)	*Neopsocopsis profunda* (= *Neoblaste schizopetala*; = *Neoblaste flavae*)
6	Clunium with posterior margin sharply convex medially (Fig. 2B); internal lobes of hypandrium distally forked (Fig. 2C)	*Neopsocopsis convexa* sp. n.
–	Clunium with posterior margin smoothly convex medially (Fig. 6B); internal lobes of hypandrium tortuous forming right-angle and distally not forked (Fig. 6C)	*Neopsocopsis flavida* (=*Blastopsocidus pini*; =*Pentablaste lanceolata*)

### Key to adult females of *Neopsocopsis*

**Table:** 

1	Subgenital plate with egg guide not sclerotized	2
–	Subgenital plate with egg guide sclerotized wholly (Fig. 2E) or at basal 1/3–1/2 (Fig. 5E)	4
2	Fore wing pale brown with anterior part dark colored; outer valve of gonapophyses with posterior lobe narrowing to internal tip. See Li 2002, pp. 1381, Fig. 1240	*Neopsocopsis minuscula*
–	Fore wing pale brown wholly; outer valve of gonapophyses with posterior lobe broad at internal tip	3
3	Subgenital plate with large membranous region anteromedially, pigment arms with small lateral expansions, widely separated; dorsal valve of gonapophyses slender. See Li 2002, pp. 1384, Fig. 1243	*Neopsocopsis longicaudata*
–	Subgenital plate with small membranous region anteromedially, pigment arms with large lateral expansions close to each other; dorsal valve of gonapophyses broad. See Li 2002, pp. 1383, Fig. 1242	*Neopsocopsis auctachila*
4	Subgenital plate with egg guide sclerotized wholly (Fig. 2E)	*Neopsocopsis convexa* sp. n.
–	Subgenital plate with egg guide sclerotized at basal 1/3–1/2	5
5	Subgenital plate with distal margin of sclerotized regions straight (Fig. 5E)	6
–	Subgenital plate with distal margin of sclerotized regions not straight (Fig. 3E)	7
6	Subgenital plate with egg guide sclerotized at basal 1/3 (Fig. 5E); internal plate with round plate surrounding spermathecal opening (Fig. 5G)	*Neopsocopsis profunda* (= *Neoblaste schizopetala*; = *Neoblaste flavae*)
–	Subgenital plate with egg guide sclerotized at basal 1/2; internal plate without round plate surrounding spermathecal opening. See Li, 2002, pp. 1385, Fig. 1244	*Neopsocopsis jinxiuica*
7	Egg guide with distal margin of sclerotized regions sharply convex (Fig. 6E)	8
–	Egg guide with distal margin of sclerotized regions concave medially, forming fork-like structure (Fig. 3E)	9
8	Small in size, forewing length about 3.1–3.7 mm; outer valve of gonapophyses with posterior lobe broad at internal tip (Fig. 6F)	*Neopsocopsis flavida* (=*Blastopsocidus pini*; =*Pentablaste lanceolata*)
–	Large in size, forewing length about 4.1 mm; outer valve of gonapophyses with posterior lobe narrowing to internal tip. See Li 2002, pp. 1386, Fig. 1245	*Neopsocopsis lushannensis*
9	Small in size, forewing length about 2.5–3.0 mm; egg guide with short neck region; subgenital plate with large membranous region anteromedially. See Yoshizawa 2010, Fig. 11	*Neopsocopsis sakishimensis*
–	Large in size, forewing length more than 3.0 mm; egg guide with relatively long neck region; subgenital plate with small membranous region anteromedially	10
10	Egg guide with fork-like sclerotized regions slightly concave; internal plate with two small sclerotized regions laterally. See Yoshizawa 2010, Fig. 13	*Neopsocopsis longiptera* (= *Pentablaste tetraedrica*)
–	Egg guide with fork-like sclerotized regions strongly concave (Fig. 3E); internal plate with sclerotized regions marginally, paired with strong processes directed laterally (Fig. 3G)	*Neopsocopsis hirticornis* (=*Pentablaste obconica*; =*Pentablaste clavata*)

## Descriptions

### Neopsocopsis convexa

Liu, Li & Liu sp. n.

urn:lsid:zoobank.org:act:DB945C8B-2D6F-4367-B9C7-15332C71F172

http://species-id.net/wiki/Neopsocopsis_convexa

Figures 1A , 2

#### Type material.

Holotype ♂: China, Yunnan Prov., Lüchun Co., Huanglianshan Natural Reserve, 5.v.2011 (LX Liu). Paratypes. China: 1♀, same date as holotype; 1♂1♀, same locality and collector as holotype, 6.v.2011; 1♀, Yunnan Prov., Jinping Co., Fenshuiling Natural Reserve, 9.v.2011 (LX Liu); 1♂1♀, same locality and collector, 10.v.2011; 2♂, Yunnan Prov., Pingbian Co., Daweishan Natural Reserve, 16.v.2006 (JX Cui); 1♂, same locality, 12.v.2006 (Y Tang); 3♂, same locality, 24.v.2009 (XS Yang); 1♂, Gansu Prov., Wenxian Co., Qiujiaba Reg., 26.vii.2011 (SP Liu).

#### Etymology.

The specific name refers to the characteristic convex-shaped posteromedian lobe of the hypandrium.

#### Diagnosis.

Medium sized psocids. Fore wing hyaline with brownish coloration; Rs and M fused for very short distance, meeting at point or connected by crossvein. Male: 8^th^ sternum strongly sclerotized and fused to hypandrium; epiproct swollen with tiny projection at middle of anterior margin; hypandrium 5-lobed with posteromedian lobe convex distaromedially, internal lobes rod-like and distally forked. Female: subgenital plate with egg guide distally round, slightly sclerotized, pigment arms forming flat V-shaped regions and expanded laterally.

#### Discription.

**Figure 1.**
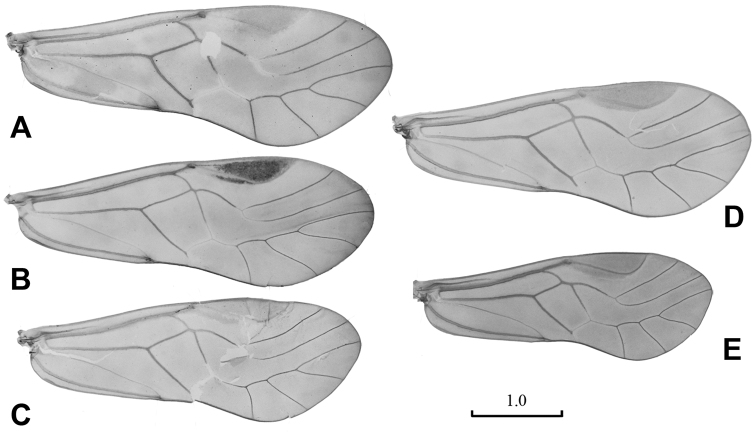
Male wings. **A** *Neopsocopsis convexa* sp. n. **B** *Neopsocopsis hirticornis* **C** *Neopsocopsis quinquedentata* **D** *Neopsocopsis profunda* **E** *Neopsocopsis flavida*. Scales in mm.

Male. Head creamy brown; compound eyes grayish black, ocelli black with grayish black ocellar field; antennae and labrum brown; maxillary palpi brown with distal segments dark colored. Thorax brown with dark brown markings on mesonotum; legs brown, with tarsi and distal part of tibia dark brown. Abdominal segments mostly creamy white, with apical regions dark brown. Fore wing (Fig. 1A) hyaline with brownish tinge, pterostigma dark brown with dark brown band along proximal margin; veins brown, except for Rs fork and M-Cu_1a_ fusion hyaline. Venation: Rs and M fused for very short distance, meeting at point or connected by crossvein; distal margin of discoidal cell straight; first and second sections of Cu_1a_ almost equal length, diverging at angle about 120°. Hind wing hyaline with brownish coloration; veins brown.

**Figure 2.**
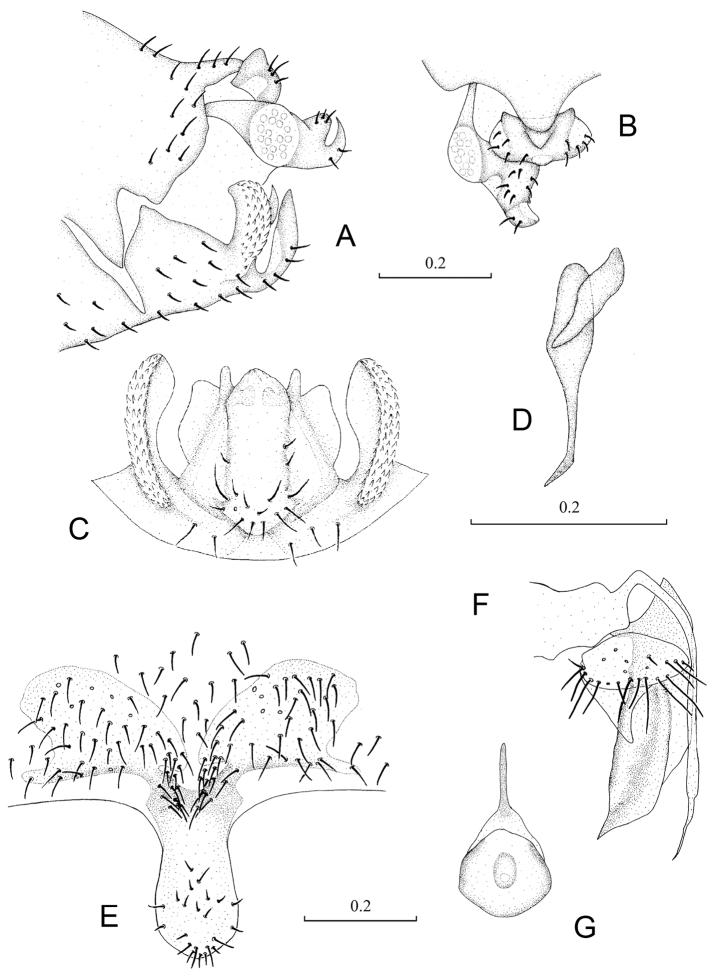
Terminalia of *Neopsocopsis convexa* sp. n.. **A** terminalia, lateral view **B** terminalia, dorsal view **C** hypandrium, posterior view **D** phallosome, lateral view **E** subgenital plate, ventral view **F** gonapophyses **G** internal plate, ventral view. Scales in mm. **AB, CD, E–G** to common scale.

Abdomen. Male genitalia: 8^th^ sternum strongly sclerotized and fused to hypandrium. Clunium (Fig. 2A) with posterior margin sharply convex medially. Epiproct (Fig. 2AB) swollen, unsclerotized medially, with tiny projection at middle of anterior margin. Paraproct (Fig. 2A) round and broadened distally. Hypandrium 5-lobed, lateral lobes carinate with outer surface covering denticles; posteromedian lobe forming dorsal-curved structure, with distal margin convex medially, basally with small membranous regions; internal lobes rod-like and distally forked. Phallosome (Fig. 2D) free posteriorly, distally broadened and paired with parameres.

Female genitalia: Subgenital plate (Fig. 2E) with egg guide round distally, invaginated basally and slightly sclerotized; pigment arms forming flat V-shaped regions and expanded laterally. Gonapophyses (Fig. 2F) with ventral valve distally tapering to slender tip; dorsal valve broad with pointed distal process; outer valve oval, with posterior lobe slender and well pointed. Internal plate (Fig. 2G) brown around spermathecal opening and marginally, with rod-like dark brown sclerotization anteriorly.

#### Measurements.

Male: Body length 2.5–3.2 mm; fore wing length 3.9–4.3 mm; hind wing length 2.9–3.6 mm. Female: Body length 3.2–3.9 mm; fore wing length 3.7–4.4 mm; hind wing length 2.6–3.2 mm.

#### Distribution.

China (Gansu, Yunnan).

#### Discussion.

The new species appears to be closely related to *Neopsocopsis hirticornis* (Reuter, 1893), *Neopsocopsis sakishimensis* (Yoshizawa, 2010) and *Neopsocopsis flavida* (Li, 1989) based on similarity of the hypandrium with posteromedian lobe convex distaromedially. However, it can be easily separated from them by the larger body size, by the structure of male clunium, and by the shape and sclerotized pattern of the female subgenital plate. The new species is distinguished from all the other *Neopsocopsis* species by the characteristic shape of the internal lobes of hypandrium.

### Neopsocopsis hirticornis

(Reuter, 1893)

http://species-id.net/wiki/Neopsocopsis_hirticornis

Figures 1B , 3

Psocus hirticornis Reuter, 1893: 42.
Neopsocopsis hirticornis (Reuter). Badonnel, 1938: 239.
Psocus bastmannianus Enderlein, 1918: 487. Synonymy: Badonnel, 1938: 239.
Neopsocus pyrenainus Badonnel, 1935: 47. Synonymy: Badonnel, 1982: 261.
Pentablaste obconica Li, 2002: 1373, syn. n.
Pentablaste clavata Li, 2002: 1368, syn. n.

#### Material examined.

*Pentablaste obconica* – Holotype ♂: China, Shanxi Prov., Wenshui Co., Guandishan Reg., 2.viii.1981 (FS Li); *Pentablaste clavata* – Holotype ♂: China, Hebei Prov., Pingquan Co., Guangtoushan Reg., 2.vii.1986 (FS Li).Other material examined.China: 1♂, Nei Mongol Aut. Reg., Alax Left. B., Helanshan Natural Reserve, 6.viii.2010 (YL Tian); 1♂, same locality, 13.viii.2010 (SG Liang); 2♂, Shanxi Prov., Wenshui Co., Guandishan Reg., 3.viii.1981 (CK Yang); 1♀, same locality, 3.viii.1981 (FS Li); 1♀, Hebei Prov., Pingquan Co., Guangtoushan Reg., 2.vii.1986 (FS Li); 1♂, Beijing M., Xiangshan Reg., 12.v.1962 (FS Li). Japan: 1♀, Kanagawa Pref., Yokohama C., Serigatani, 7.iv.2011 (Y Hoshino); 2♀, same locality and collector, 12.iv.2011.

#### Redescription.

Male. Head creamy brown, with dark brown markings; compound eyes grayish black, ocelli black with grayish black ocellar field; antennae, labrum and maxillary palpi brown. Thorax brown with dark brown spots; legs brown, with band of dark brown marking on femur, tarsi and distal part of tibia dark brown. Fore wing (Fig. 1B) hyaline with brownish tinge, pterostigma dark brown with dark brown band along proximal margin; veins brown, except for Rs fork and M-Cu_1a_ fusion hyaline. Venation: Rs and M fused for very short distance; distal margin of discoidal cell straight; first and second sections of Cu_1a_ almost equal length, diverging at angle about 120°. Hind wing hyaline; veins brown.

**Figure 3.**
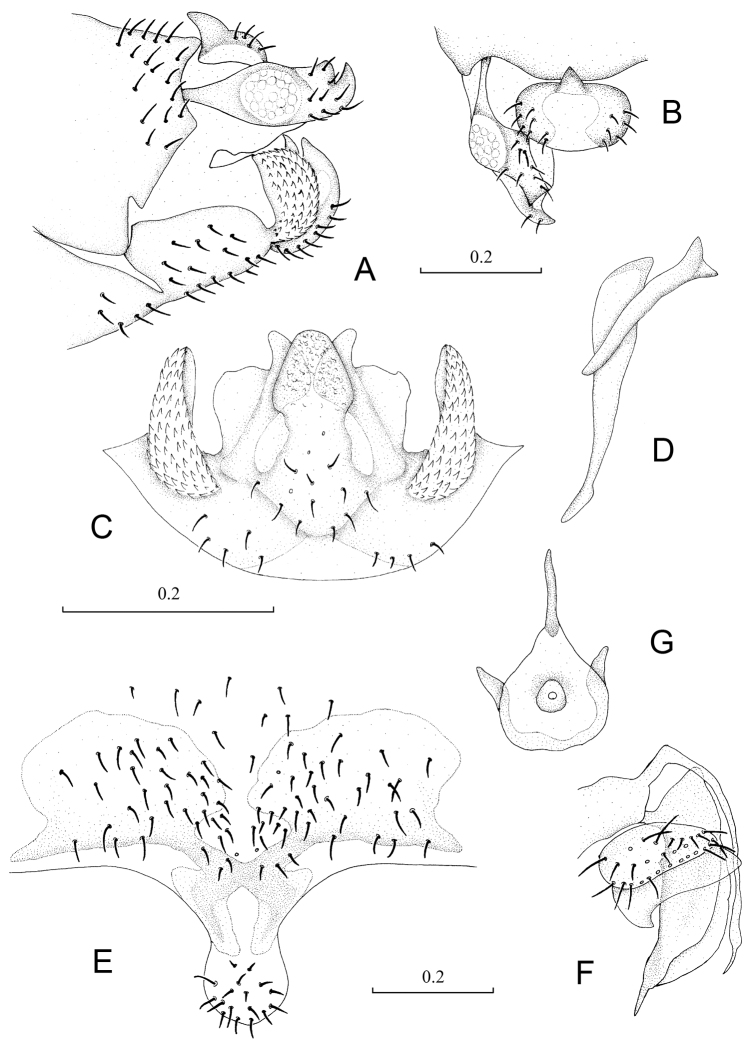
Terminalia of *Neopsocopsis hirticornis*. **A** terminalia, lateral view **B** terminalia, dorsal view **C** hypandrium, posterior view **D** phallosome, lateral view **E** subgenital plate, ventral view **F** gonapophyses **G** internal plate, ventral view. Scales in mm. **AB, CD, E–G** to common scale.

Abdomen. Male genitalia: 8^th^ sternum strongly sclerotized and fused to hypandrium. Clunium (Fig. 3A) with posterior margin convex medially and invaginated bilaterally. Epiproct (Fig. 3AB) swollen, unsclerotized medially, with sharp projection at middle of anterior margin. Paraproct (Fig. 3A) round and broadened distally. Hypandrium (Fig. 3C) 5-lobed, lateral lobes carinate with outer surface covering denticles; posteromedian lobe forming dorsal-curved structure, with distal margin smoothly round, basally with fan-shaped membranous regions; internal lobes well developed, with distal part crescent-like and directed dorsolaterally. Phallosome (Fig. 3D) free posteriorly, distally broadened and paired with parameres.

Female genitalia: Subgenital plate (Fig. 3E) with egg guide round distally, basally invaginated; pigment arms forming flat V-shaped regions and expanded laterally, posteriorly forked. Gonapophyses (Fig. 3F) with ventral valve distally tapering to slender tip; dorsal valve broad with pointed distal process; outer valve oval, with posterior lobe broad and well pointed. Internal plate (Fig. 3G) with brown coloration around spermathecal opening and marginally, with rod-like dark brown sclerotization anteriorly.

#### Measurements.

Male: Body length 2.4–2.9 mm; fore wing length 3.2–4.5 mm; hind wing length 2.5–3.3 mm. Female: Body length 3.0–3.5 mm; fore wing length 3.7–4.5 mm; hind wing length 3.0–3.6 mm.

#### Distribution.

China (Beijing, Gansu, Hebei, Hubei, Hunan, Jilin, Nei Mongol, Ningxia, Shanxi, Zhejiang: new distributional record); Finland; France; Germany; Hungary; Italy; Japan (new distributional record) ; Macedonia; Mongolia; Romania; Russia; Serbia; Spain; Sweden; Switzerland.

#### Discussion.

*Pentablaste clavata* was described by Li (2002) based on one male and one female from Hebei, with the characters of the fore wing Sc ending at R and the crossvein Rs-M. *Pentablaste obconica* is the type species of Li’s genus *Pentablaste*, which is the most widely distributed species of China. *Neopsocopsis hirticornis* is the type species of *Neopsocopsis*, distributed mainly in Europe. After reexamining all the specimens, we found they all have similar genitalia, but the crossvein Rs-M is not a stable character to distinguish *Pentablaste clavata* from the others. Thus we consider *Pentablaste obconica* and *Pentablaste clavata* be new junior synonyms of *Neopsocopsis hirticornis*. Females of *Neopsocopsis hirticornis* are mostly brachypterous (Vishnyakova 1986; Lienhard 1998), but a few macropterous females have also been recorded (Günther 1980, 1991; Hedström 1989). Females collected in Japan (new distributional record) are all brachypterous, but those from China are all macropterous. Therefore, there might be some genetic differences between Chinese and other populations of *Neopsocopsis hirticornis* but, in the absence of more distinct differences, we treat them as a single species. This species is similar to *Neopsocopsis sakishimensis* Yoshizawa, 2010 from Japan, however *Neopsocopsis hirticornis* can be distinguished by the larger body size and by the genitalic characters.

### Neopsocopsis longiptera

Vishnyakova, 1986

http://species-id.net/wiki/Neopsocopsis_longiptera

Neopsocopsis longiptera Vishnyakova, 1986: 350.
Pentablaste tetraedrica Li, 2002: 1371, syn. n.

#### Material examined.

*Pentablaste tetraedrica* – Holotype ♂: China, Hebei Prov., Pingquan Co., Guangtoushan Reg., 2.vii.1986 (FS Li). Other material examined.China: 2♂3♀, same locality and collector, 2.vii.1986; Japan: 1♂1♀, Fukuoka Pref., Hisayama C., Yamada, 5.vi.1994 (K Yoshizawa).

#### Distribution.

China (Hebei: new distributional record); Russia; Japan.

#### Discussion.

*Pentablaste tetraedrica* was described based on 3 males and 3 females from China. The species is distinguished from other Chinese species based on the character of the hypandrium posteromedian lobe lacking apically horn-like processes (Li, 2002). *Neopsocopsis longiptera* was described based on the specimens from the Russian Far East and differed from *Neopsocopsis hirticornis* (Reuter, 1893) in having a macropterous female and a larger male IO/D (Vishnyakova, 1986). After reexamining the two specimens, we found the main characters of the wings and genitalia are nearly identical. Thus we consider *Pentablaste tetraedrica* to be a new synonym of *Neopsocopsis longiptera*.

### Neopsocopsis quinquedentata

(Li & Yang, 1988)

http://species-id.net/wiki/Neopsocopsis_quinquedentata

Figures 1C , 4

Blastopsocidus quinquedentata Li & Yang, 1988: 79.
Neopsocopsis quinquedentata (Li & Yang). Yoshizawa 2010: 36.

#### Material examined.

Holotype ♂: China, Guizhou Prov., Jiangkou Co., Fanjingshan Natural Reserve, 27.vii.1983 (FS Li). Other material examined. China: 1♂, Guizhou Prov., Leishan Co., Leigongshan Natural Reserve, 14.iv.2005 (Y Tang).

#### Redescription.

Male. Head creamy brown; compound eyes grayish black, ocelli black with grayish black ocellar field; antennae and labrum brown, maxillary palpi brown with apical segment lighter. Thorax brown with dark brown spots; legs brown, with band of dark brown marking on femur, tarsi and distal part of tibia dark colored. Fore wing (Fig. 1C) hyaline with light brownish tinge, pterostigma brown; veins brown, except for Rs fork and M-Cu_1a_ fusion hyaline. Venation: Rs and M fused for very short distance; distal margin of discoidal cell straight; first section of Cu_1a_ shorter than the second section, diverging at angle about 120°. Hind wing hyaline; veins brown.

**Figure 4.**
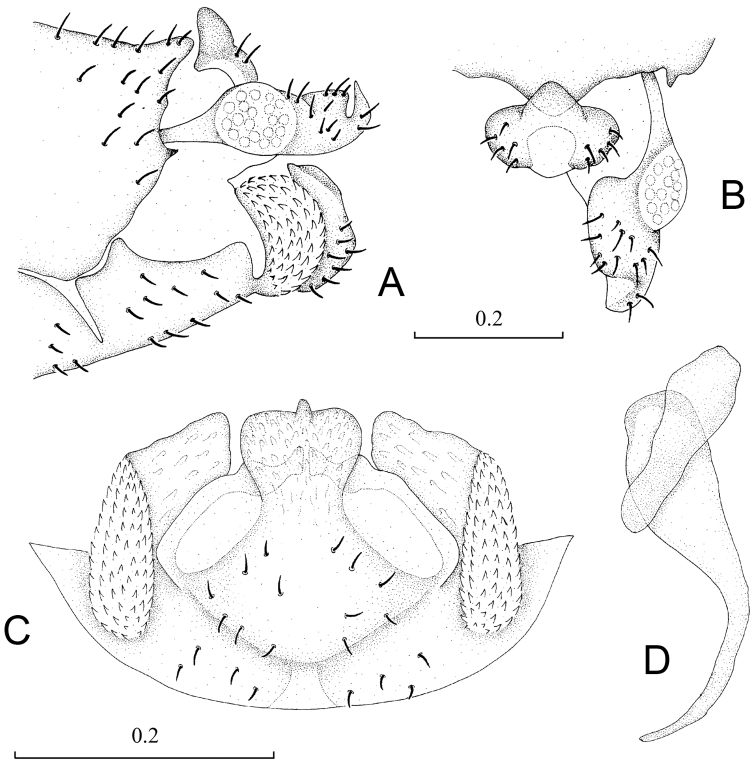
Terminalia of *Neopsocopsis quinquedentata*. **A** terminalia, lateral view **B** terminalia, dorsal view **C** hypandrium, posterior view **D** phallosome, lateral view Scales in mm. **AB, CD** to common scale.

Abdomen. Male genitalia: 8^th^ sternum strongly sclerotized and fused to hypandrium. Clunium (Fig. 4A) with posterior margin sharply convex medially and with tiny projection bilaterally. Epiproct (Fig. 4AB) swollen, unsclerotized medially, with moderate projection at middle of anterior margin. Paraproct (Fig. 4A) round and broadened distally. Hypandrium (Fig. 4C) 5-lobed, lateral lobes carinate with anterior part long and curved anteromedially, outer surface covering denticles; posteromedian lobe forming dorsal-curved structure, with distal margin almost straight and with tiny projection medially, basally with small membranous regions; internal lobes not well developed, much smaller than posteromedian lobe and distally bud-like. Phallosome (Fig. 4D) free posteriorly, distally broadened and paired with parameres.

Female unknown.

#### Measurements.

Male: Body length 1.9–2.3 mm; fore wing length 4.0–4.1 mm; hind wing length 2.9–3.1 mm.

#### Distribution.

China (Guizhou).

#### Discussion.

*Neopsocopsis quinquedentata* was described based on one male from Guizhou, with the character of fore wing Rs-M fusion. It can be separated from other species by the hypandrial posteromedian lobe with projection medially, and by the characteristic structures of internal lobes and parameres.

### Neopsocopsis profunda

(Li, 1995)

http://species-id.net/wiki/Neopsocopsis_profunda

Figures 1D , 5

Neoblaste profunda Li 1995: 186.
Neopsocopsis profunda (Li). Yoshizawa 2010:35.
Neoblaste schizopetala Li 1997: 488, syn. n.
Neoblaste flavae Li 1995: 187, syn. n.

#### Material examined.

*Neoblaste profunda* – Holotype ♂: China, Zhejiang Prov., Qingyuan Co., Baishanzu Natural Reserve, 3.x.1993 (H Wu). *Neoblaste schizopetala* – Holotype ♂: China, Chongqing M., Fengdu Co., Shiping Reg., 5.x.1994 (FS Li). *Neoblaste flavae* – Holotype ♀: China, Zhejiang Prov., Qingyuan Co., Baishanzu Natural Reserve, 27.x.1993 (H Wu). Other material examined. China: 1♀3♂, Zhejiang Prov., Qingyuan Co., Baishanzu Natural Reserve, 20.xi.1993 (H Wu); 1♀, same locality and collector, 3.x.1993; 2♀, same locality and collector, 27.x.1993; 1♂, Chongqing M., Fengdu Co., Guicheng Reg., 4.x.1994 (FS Li); 1♀, Chongqing M., Fengdu Co., Shiping Reg., 5.x.1994 (FS Li); 1♂, Hubei Prov., Xingshan Co., Longmenhe Reg., 12.ix.1994 (FS Li); 1♀, Henan Prov., Luanchuan Co., Longyuwan Reg., 7.viii.2008 (WH Li).

#### Redescription.

Male.Head yellowish, with brown markings; compound eyes grayish black, ocelli black with grayish black ocellar field; antennae and labrum brown, maxillary palpi brown with distal segments dark colored. Thorax brown with dark brown spots; legs pale brown. Fore wing (Fig. 1D) hyaline with yellowish tinge, pterostigma and veins brown, except for Rs fork and M-Cu_1a_ fusion hyaline. Venation: Rs and M connected by short crossvein or meeting at point; distal margin of discoidal cell straight; first and second sections of Cu_1a_ almost equal length, diverging at angle about 120°. Hind wing hyaline; veins brown.

**Figure 5.**
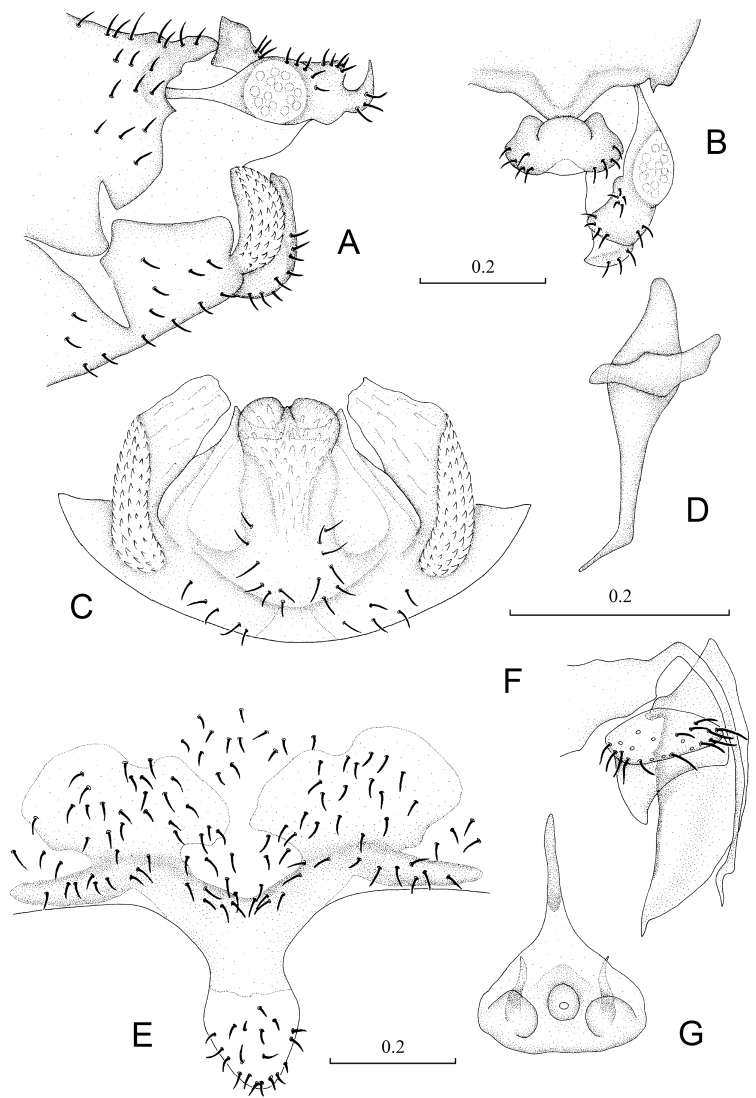
Terminalia of *Neopsocopsis profunda*. **A** terminalia, lateral view **B** terminalia, dorsal view **C** hypandrium, posterior view **D** phallosome, lateral view **E** subgenital plate, ventral view **F** gonapophyses **G** internal plate, ventral view. Scales in mm. **AB, CD, E–G** to common scale.

Abdomen. Male genitalia: 8^th^ sternum strongly sclerotized and fused to hypandrium. Clunium (Fig. 5A) with posterior margin convex medially and with slight invagination bilaterally. Epiproct (Fig. 5AB) swollen, unsclerotized medially, with round projection at middle of anterior margin. Paraproct (Fig. 5A) round and broadened distally. Hypandrium (Fig. 5C) 5-lobed, lateral lobes carintae with anterior part long and curved anteromedially, outer surface covering denticles; posteromedian lobe forming dorsal-curved structure, with distal margin concave and with tiny projection medially, basally with small membranous regions; internal lobes rod-like and distally forked. Phallosome (Fig. 5D) free posteriorly, distally broadened and paired with parameres.

Female genitalia: Subgenital plate (Fig. 5E) with egg guide round with slightly narrowed margins distally, basally invaginated and sclerotized at basal 1/3; pigment arms forming flat V-shaped regions and expanded laterally. Gonapophyses (Fig. 5F) with ventral valve distally tapering to slender tip; dorsal valve broad with pointed distal process; outer valve oval, with posterior lobe broad and well pointed. Internal plate (Fig. 5G) with brown coloration around spermathecal opening and marginally, with rod-like dark brown sclerotization anteriorly.

#### Measurements.

Male: Body length 3.0–3.2 mm; fore wing length 3.7–4.1 mm; hind wing length 2.9–3.1 mm. Female: Body length 3.1–3.5 mm; fore wing length 3.8–4.6 mm; hind wing length 2.8–3.4 mm.

#### Distribution.

China (Chongqing, Henan, Hubei, Zhejiang).

#### Discussion.

*Neoblaste profunda* was described by Li (1995) based on specimens from Zhejiang, and *Neoblaste schizopetala* was described on the basis of one male and one female from Chongqing (Li, 1997). Li pointed out that both species were very similar to *Neopsocopsis quinquedentata* (Li & Yang, 1988), and could be differentiated by characters of the male hypandrium and phallosome. *Neoblaste flavae* was described based on a single female specimen from Zhejiang, which was collected with a female of *Neopsocopsis profunda* and differed from the latter by larger body size and the form of the internal plate (Li 2002). After reexamining all the species, we found there are only minor differences between these three species, e.g. the color markings in fore wings and pigment patterns of the female subgenital plate. Therefore, we consider *Neoblaste schizopetala* and *Neoblaste flavae* to be two new synonyms of *Neopsocopsis profunda*. The species can be separated from the other species by the following features: hypandrial posteromedian lobe concave at distal margin with tiny projection medially, subgenital plate with egg guide sharply round distally and sclerotized at basal 1/3.

### Neopsocopsis flavida

(Li, 1989)

http://species-id.net/wiki/Neopsocopsis_flavida

Figures 1E , 6

Blastopsocidus flavidus Li 1989: 46.
Neopsocopsis flavida (Li). Yoshizawa 2010: 35.
Blastopsocidus pini Li 1990: 5, syn. n.
Pentablaste lanceolata Li 2002: 1377, syn. n.

#### Material examined.

*Blastopsocidus flavidus* – Holotype ♂: China, Guizhou Prov., Guiyang C., Huaxi D., 9.vi.1981 (FS Li).*Blastopsocidus pini* – Holotype ♂: China, Guizhou Prov., Guiyang C., Bagongli Reg., 21.viii.1988 (FS Li).*Pentablaste lanceolata* – Holotype ♂: Guizhou Prov., Guiyang C., Bagongli Reg., 21.viii.1988 (FS Li).Other material examined.China: 13♀22♂, Guizhou Prov., Guiyang C., Huaxi D., 9.vi.1981 (FS Li); 1♂, same locality and collector, 27.v.1981; 1♂, same locality and collector, 28.v.1981; 7♀3♂, Guizhou Prov., Guiyang C., Bagongli Reg., 21.viii.1988 (FS Li); 1♂, Guizhou Prov., Leishan Co., Leigongshan Natural Reserve, 16.iv.2005 (Y Tang); 1♂, Fujian Prov., Nanping C., Wuyishan Reg., 4.vii.2009 (XS Yang); 4♀6♂, Hunan Prov., Hengyang C., Nanyue D., 20.vi.1963 (CK Yang); 1♂, Shanxi Prov., Wutai Co., Wutaishan Reg., 24.vii.1981 (FS Li); 1♂, Guangxi Prov., Longzhou Co., Nonggang Natural Reserve, 21.v.1982 (CK Yang); 1♀, Jiangxi Prov., Jiujiang C., Lushan Reg., 6.ix.1959 (CK Yang); 1♀1♂, Shaanxi Prov., Pingxiang C., Wugongshan Reg., 22.vii.1962 (CK Yang); 3♀3♂, Anhui Prov., Huangshan C., Huangshan Reg., 18.vii.1977 (FS Li).

#### Redescription.

Male. Head brownish, with dark brown markings; compound eyes grayish black, ocelli black with grayish black ocellar field; antennae and labrum brown, maxillary palpi brown with distal segments dark colored. Thorax brown with dark brown spots dorsally; legs pale brown, with tarsi dark brown. Fore wing (Fig. 1E) hyaline with yellowish tinge, pterostigma and veins brown, except for Rs fork and M-Cu_1a_ fusion hyaline. Venation: Rs and M fused for short distance; distal margin of discoidal cell straight; first section of Cu_1a_ little longer than the second section, diverging at angle about 120°. Hind wing hyaline; veins brown.

**Figure 6.**
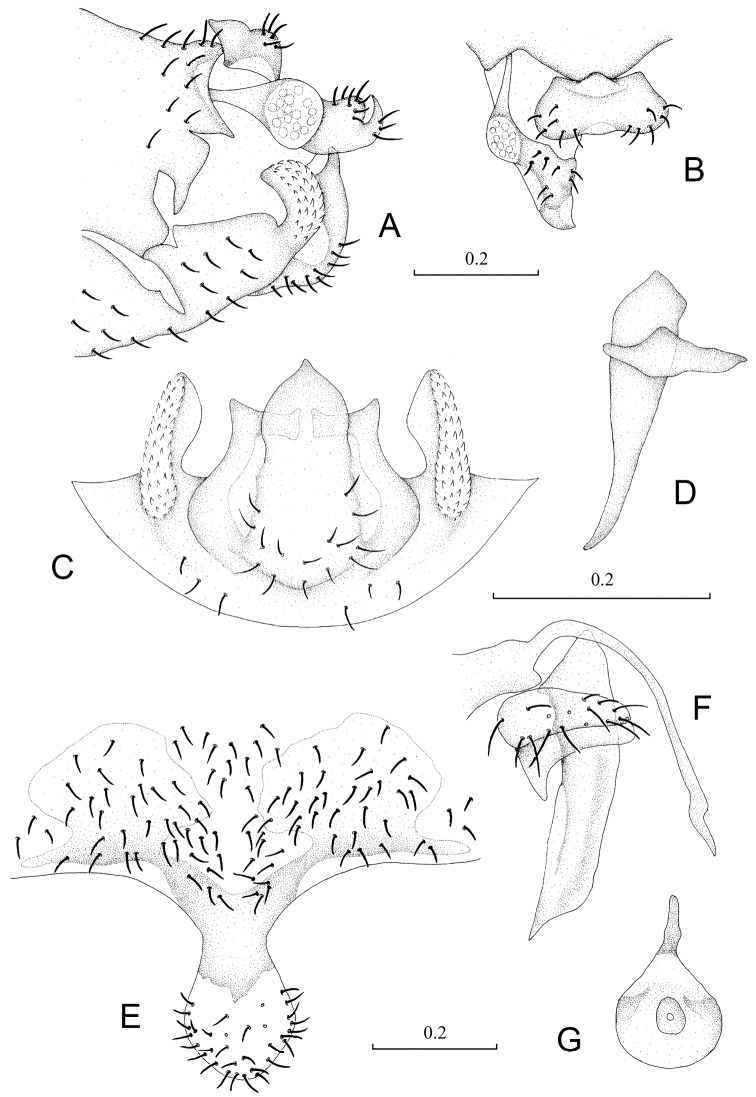
Terminalia of *Neopsocopsis flavida*. **A** terminalia, lateral view **B** terminalia, dorsal view **C** hypandrium, posterior view **D** phallosome, lateral view **E** subgenital plate, ventral view **F** gonapophyses **G** internal plate, ventral view. Scales in mm. **AB, CD, E–G** to common scale.

Abdomen. Male genitalia: 8^th^ sternum strongly sclerotized and fused to hypandrium. Clunium (Fig. 6A) with posterior margin convex medially. Epiproct (Fig. 6AB) swollen, unsclerotized medially, with round projection at middle of anterior margin. Paraproct broad. Hypandrium (Fig. 6C) 5-lobed, lateral lobes carinate with outer surface covering denticles; posteromedian lobe forming dorsal-curved structure, with distal margin convex tapering to point; internal lobes tortuous forming right-angle and distally not forked. Phallosome (Fig. 6D) free posteriorly, distally broadened and paired with parameres.

Female genitalia: Subgenital plate (Fig. 6E) with egg guide round distally, basally invaginated and sclerotized at basal 1/3; pigment arms forming flat V-shaped regions and expanded laterally. Gonapophyses (Fig. 6F) with ventral valve distally tapering to slender tip; dorsal valve long and narrow with pointed distal process; outer valve oval, with posterior lobe narrow and well pointed. Internal plate (Fig. 6G) with brown coloration around spermathecal opening and marginally, with rod-like dark brown sclerotization anteriorly.

#### Measurements.

Male: Body length 2.1–2.9 mm; fore wing length 2.6–3.6 mm; hind wing length 2.0–2.7 mm. Female: Body length 2.6–3.3 mm; fore wing length 3.1–3.8 mm; hind wing length 2.4–2.9 mm.

#### Distribution.

China (Anhui, Fujian, Guangxi, Guizhou, Hunan, Jiangxi, Shaanxi, Shanxi).

#### Discussion.

These three species were very similar according to Li (1990, 2002). *Neopsocopsis flavida* was described based on the holotype specimen from Huaxi (Guiyang), with distribution range in central and southeast China. *Blastopsocidus pini* was described based on specimens from Bagongli (Guiyang), and could be differentiated from *Neopsocopsis flavida* by the fore wing veins M<M_1+2_ and by a smaller outer valve of the gonapophyses (Li, 1990). In 2002, Li described *Pentablaste lanceolata* based on 3 males which were formerly named under *Blastopsocidus pini*, with different character states of the postclypeus, paraproct and parameres. By our reexamination of all these species, there are only minor differences among *Neopsocopsis flavida*, *Blastopsocidus pini* and *Pentablaste lanceolata*, and it is hardly possible to distinguish them from each other by the genitalic characters. Therefore, we consider *Blastopsocidus pini* and *Pentablaste lanceolata* to be new junior synonyms of *Neopsocopsis flavida*. The species is distinguished from the other species by the posteromedian lobe of the male hypandrium convex tapering to a point, by the pigmented pattern of the female subgenital plate and by the narrow dorsal valve of the gonapophyses.

## Supplementary Material

XML Treatment for Neopsocopsis

XML Treatment for Neopsocopsis convexa

XML Treatment for Neopsocopsis hirticornis

XML Treatment for Neopsocopsis longiptera

XML Treatment for Neopsocopsis quinquedentata

XML Treatment for Neopsocopsis profunda

XML Treatment for Neopsocopsis flavida
